# Recommendations for Probiotic Use in Humans—A 2014 Update

**DOI:** 10.3390/ph7100999

**Published:** 2014-10-10

**Authors:** Martin H. Floch

**Affiliations:** Yale University School of Medicine, 333 Cedar Street, 1080 LMP, New Haven, CT 06850, USA; E-Mail: martin.floch@yale.edu; Tel.: 203-855-0620; Fax: 203-227-1442

**Keywords:** probiotics, antibiotic diarrheas, IBS, IBD

## Abstract

Probiotics have gained worldwide use during the last two decades. However, which probiotic to use in which clinical condition has remained confusing in some clinical conditions. We convened a workshop at Yale in conjunction with Harvard in 2005, inviting a spectrum of probiotic authorities to discuss and reach conclusions on recommendations for use in common clinical conditions; the workshop was reconvened again in 2008 and in 2011. Each time the group of authorities was enlarged and varied depending on research studies. This article lists the recommendations updated from 2011 and is amended to bring it up to date in childhood and adult diarrhea, antibiotic-associated diarrhea, necrotizing enterocolitis, inflammatory bowel disorders, irritable bowel syndrome, allergic disorders, and radiation enteritis pending our 4th Triennial Yale/Harvard workshop to be convened in 2015.

## 1. Introduction

The burgeoning use of probiotics has proliferated during the past two decades [1]. Although history has shown us that probiotics were part of foods in the past, they have begun to be used therapeutically by clinicians in regular diets [2]. When the popularity of probiotics became apparent, we convened a workshop meeting at Yale University, which included experts in the field of probiotic organisms, to made recommendations for their clinical use. This first workshop meeting occurred in 2006, and we reconvened two years later with some of the same experts and leading authorities in the field, and the last meeting occurred in 2011 [3,4,5]. The amount of literature has exploded, and many more opinions have been expressed [6]. It is our intention to have a 4th Yale workshop meeting to bring the clinical recommendations up to date in 2015.

## 2. Necrotizing Enterocolitis

Table 1 lists the recommendations made in 2011 by the expert Yale University panel [5]. When we reviewed these, we added the important literature from the past few years. It is clear that necrotizing enterocolitis is one of the most hazardous conditions in any stage of life [7]. Fortunately, when correctly diagnosed, it has been controlled with the use of bovine lactoferrin in combination with probiotic *Lactobacillus rhamnosus GG* [7,8].

**Table 1:** Recommendations for Probiotic Use—Update 2011. Data from [5]. Copyright (2011) Lippincott Williams & Wilkins.

Clinical Condition		Effectiveness	Specific Strain of Organism & Strain References	Analysis References
**Diarrhea**				
Infectious childhood—treatment		A	*S. boulardii* [9], *LGG* [10,11], *L. reuteri* SD2112 [12]	[9,10,11,12]
Prevention of AAD		A	*S. boulardii* [14], *LGG* [15], combination. of *L. casei* DN114 G01, *L. bulgaricus*, snf *S. thermophiles* [16]	[14,16]
Prevention of recurrent CDAD		B/C	*S. boulardii* [17], *LGG* [13], Bacteriotherapy [18]	[17]
Prevention of CDAD		B/C	*LGG* [17], *S. boulardii* [13]	[13,17]
**IBD**				
Pouchitis				
	Preventing and maintaining remission	A	VSL#3 [19,20,21]	[19,20,21]
	Induce remission	C	VSL#3 [22]	[22]
Ulcerative colitis				
	Inducing remission	B	*E. coli* Nissle [23], VSL#3 [24]	[23,24]
	Maintenance	A	*E. coli* Nissle, VSL#3 [25,26]	[25,26]
Crohn’s		C	*E. coli* Nissle [27], *S. boulardii* [28], *LGG* [29]	[26,28,29]
**IBS**				
		B	*B. infantis* B5624[30,31], VSL#3 [32,33,34]	[30,31,32,33,34] **
		C	*Bifidobacterium animalis* [35]	[35]
			*L. plantarum* 299V [36]	[36]
**Necrotizing Enterocolitis**				
		B	*Lactobacillus acidophilus* NCDO1748 [7,37] and *Bifidobacterium bifidium* NCDO1453 [8]	[37]
Recommendations from 2008 *				
**Immune Response**				
		A	*LGG*, *L. acidophilus* LAFT1, *L. plantarum*, *Bifidobacterium lactis*, *Lactobacillus johnsonii*	[27,38]
**Allergy**				
Atopic eczema associated with cow’s milk allergy				
Treatment		A	*LGG*, *B. lactis* [39]	[39]
Prevention		A	*LGG*, B. lactis [39]	[39]
**Radiation Enteritis**				
		C	VSL#3 [38], *L. acidophilus* [27]	[27,38]
**Vaginosis and Vaginitis **				
		C	*L. acidophilus* [40], *Lactobacillus rhamnosus* GR-1 [41], *L. reuteri* RC14 [42]	[40,41,42]

AAD indicates antibiotic-associated diarrhea; IBD, inflammatory bowel disease; IBS, irritable bowel syndrome; CDAD, *Clostridium difficile*-associated diarrhea; LGG, *Lactobacillus GG*. * Check 2008 references for further elaboration on strains used and their availability. ** Reference [39] was made available after the workshop meeting on April 8, 2011 but believed to be significant enough to qualify this probiotic to be in a B category. “A” recommendation is based on strong, positive, well-conducted, controlled studies in the primary literature, not abstract form. “B” recommendation is based on positive, controlled studies but the presence of some negative studies. “C” recommendation is based on some positive studies but clearly an inadequate amount of work to establish the certainty of “A” or “B”.

## 3. Childhood Diarrhea

The microbiota is maintained in a stable ecology, and it appears that probiotics are very helpful in shortening the course of acute gastroenteritis diarrhea [9,10,11,12]. There are numerous studies to establish this. It is clear that starting *Saccharomyces boulardii*, *LGG*, or strains of *Lactobacillus reuteri* are extremely helpful in shortening the course of the diarrhea [9,10,11,12].

## 4. Adult Diarrhea

All of the more recent studies, particularly the one done by Hickson and associates in England, found that *Lactobacillus casei*, *L. bulgaricus*, and *S. thermophilus* were capable in reducing the incidence of diarrhea as well as having some effect on *Clostridium difficile*. This study also showed the potential to decrease morbidity, healthcare costs, and mortality in patients over the age of 50 [16].

## 5. Antibiotic-associated Diarrhea

Review of the modern literature in our Yale workshops [3,4,5] and by the most recent Goldenberg Cochrane Database System Review [43] clearly indicate that probiotics are helpful in the prevention of *C. difficile-*associated diarrhea in both adults and children. This literature indicates *S. boulardii*, *LGG*, in combinations [5] are helpful in accomplishing this outcome in antibiotic-associated diarrhea. A review of the literature by experts indicates that antibiotic-associated diarrhea can be prevented by the use of numerous organisms [43].

This entire issue has been clouded by the development of fecal microbial transplantation (FMT). It is clear now that FMT can cure and prevent resistant *C. difficile* infection [44]; the literature on this is extensive [45,46,47,48]. Furthermore, there are now centers to transplant volunteer specimens [23]. They are even suggestions this become a routine or government approved product that can be used for the cure and prevention of resistant *C. difficile* diarrhea. Surely, more will be forthcoming on this in the coming years.

## 6. Inflammatory Bowel Disease

A review of the literature reveals that pouchitis can be prevented, and remission can be maintained with the use of the probiotic VSL#3 [19,20,21]. Although ulcerative colitis is universally treated with corticosteroids and other agents, probiotics have some successful therapy reports. Review of the literature reveals that there is a dysbiosis that occurs in simple ulcerative colitis [49,50,51]. This is the same dysbiosis that is treated with FMT. Both previous analysis of the literature [5] has shown that ulcerative colitis could be placed into remission by several probiotics, including *Escherichia coli* Nissle, *S. boulardii* and *LGG*; however, VSL#3 has been the most successful [24,25]. These are limited studies, and most clinicians still prefer the use of more acute, aggressive agents [24,25]. However, there are scattered reports, such as that of Brace and colleagues, who have shown that the dysbiosis, which is seen with *C. difficile,* can be corrected in selective ulcerative colitis patients and reduce symptom control [50]. Shen and colleagues conducted a careful analysis of 23 randomized controlled trials with a total of 1763 cases and found from their analysis that VSL#3 was the most effective in ulcerative colitis, but there is much less of an effect in Crohn’s disease [51]. Review of the literature confirms that Crohn’s disease does not respond as well as ulcerative colitis [52].

## 7. Irritable Bowel Syndrome (IBS)

IBS is a major international clinical problem. Review of the modern literature reveals that *Bifidobacterium infantis* B5624 evaluated in Ireland has given the best reports for relief of symptoms, but most investigators do not give it an “A” rating, but more like a “B” rating [30,31]. The same investigators did find that the probiotic had immunomodulary effects on the microbiota in humans, and it did seem to extend from the mucosal system to the systemic immune system. [31]. Although this probiotic did have these immune effects, it still only had a limited effect on IBS. The IBS literature reveals human effects by *Lactobacillus plantarum* and *Bifidobacterium animalis* on IBS [35,36]. Cha and colleagues conducted another study on IBS in which 50 patients with diarrhea IBS were randomized into placebo where probiotics mixtures, including *Lactobacillus acidophilus*, *L. plantarum*, *Lactobacillus rhamnosus*, *Bifidobacterium breve*, *Bifidobacterium lactis*, *Bifidobacterium longum*, and *Streptococcus thermophilus* were used. The treatment was daily for 8 weeks, and the outcome was adequate relief of overall IBS symptoms [53]. Secondary quality of life symptoms were also studied. There was some stabilization of the intestinal microbiota in that there were some higher concordance rates between bacterial compositions before and after treatment, but we must remember that this was a small group of patients with a limited effect. Effect on probiotics on IBS still remains controversial. At this point in time, we can recommend *B. infantis* B5624, and a large group of other organisms is mentioned in the references [30,31,35,36].

## 8. Allergic Disorders

Table 1 lists the organisms that are effective on the immune response in allergic condition. We definitely have a large number of organisms, and they should be used in accordance with the strains recommended in articles [39,54].

## 9. Radiation Enteritis

Table 1 lists fair results in treating radiation enteritis. Since those reviews, there has been extensive literature discussion and reviews by Hakansson and Molin [54]. All possible probiotic organisms that have been tried are listed extensively, but there is no one set protocol which is recommended to prevent radiation diarrhea. The clinician should carefully review the literature and then decide which should be used in which particular post-radiation treatment [27,38].

Table 1 outlines guidelines for probiotic use. It is updated in this manuscript from the previous report [4]. The recommendations for these guidelines are based on the discussions at the workshop and in the literature.

The field of probiotics and symbiotics has now grown tremendously. The interested clinician should search the literature, and use the probiotic in the particular clinical situation that is needed.
